# Genetic diversity of a recovering European roller (*Coracias garrulus*) population from Serbia

**DOI:** 10.1371/journal.pone.0308066

**Published:** 2024-08-08

**Authors:** Ivana Matić, Nevena Veličković, Dimitrije Radišić, Lea Milinski, Mihajla Djan, Milomir Stefanović

**Affiliations:** Faculty of Sciences, Department of Biology and Ecology, University of Novi Sad, Novi Sad, Serbia; University of Ghana, GHANA

## Abstract

The European Roller (*Coracias garrulus*), a long-distance migratory bird, faced a considerable decline in breeding pairs throughout Europe at the end of the 20th century. Due to conservation efforts and the installation of nesting boxes, the population of the European Roller in Serbia has made a remarkable recovery. Here, we used the variability of nucleotide sequences of the mitochondrial DNA (mtDNA) control region and 10 microsatellite loci to assess the genetic diversity and structuring, phylogeographic patterns and demographic history of this species using 224 individuals from Serbia. Our results showed moderate level of genetic diversity (H_O_ = 0.392) and a slightly elevated level of inbreeding and homozygosity (F_IS_ = 0.393). Genetic structuring based on microsatellite data indicated three genetic clusters, but without a clear spatial pattern. High haplotype diversity (Hd = 0.987) of the mtDNA control region sequences was detected, and neutrality tests indicated a recent demographic expansion. The phylogeographic analysis, which also included previously published sequences of the mtDNA control region, supported the subdivision into two distinct European and Asian haplogroups (Φ_ST_ = 0.712). However, the results of our study showed that a larger number of haplotypes sampled in Serbia are clustered in the Asian haplogroup as compared to previous studies, indicating a historically continuous distribution of this species and possibly a wider distribution of the subspecies *Coracias garrulus semenovwi*. Our results suggest that the European Roller population in Serbia is genetically stable, with no evidence of recent bottlenecks, and emphasize the importance of artificial nest boxes for promoting and maintaining population dynamics of European Rollers.

## Introduction

The European roller (*Coracias garrulus* Linnaeus, 1758; hereafter: roller) is a long-distance migratory bird species, wintering in sub-Saharan Africa. Rollers are insectivorous, secondary cavity nesters, with two distinguished subspecies: the nominal subspecies (*Coracias garrulus garrulus* Linnaeus, 1758) which breeds in northwest Africa, Europe, Asia Minor and southwest Siberia, and *Coracias garrulus semenowi* [1] which breeds in Iraq, Iran, east to Kashmir, north to Turkmenistan, southern Kazakhstan and northwestern China [2,3]. Over the past decades, many roller populations in Europe have faced a drastic decline in the number of breeding pairs, particularly in northern and central Europe where it even went extinct in several countries [4]. Land use changes, driven by agricultural intensification and modernization, which resulted in the reduction of natural breeding habitats, nesting cavities, and prey availability, are among the most significant factors that have led to the decline of the roller populations in Serbia, and throughout Europe [5,6]. In the 1950s, the population size of rollers in Serbia (Vojvodina province) was stable, but just as was the case globally, there was a sharp decline between 1980 and 1990, and in 1996 only about ten breeding pairs were detected in Vojvodina province [7,8]. This region once harbored one of the most critical local populations of rollers, particularly concentrated in northern Bačka and Banat. In the early 1990s, breeding of rollers was observed only in a few locations in Banat [9]. Later, in the early 2000s, the population south of the Sava and Danube rivers numbered around 40–45 breeding pairs [10]. The sparse sightings and field observations in the early 1990s underline this worrying local extinction trend, not only in Serbia, but also in other central and northern European regions. However, conservation efforts through the installation of wooden nest boxes across Vojvodina province have contributed to the recovery of the roller population increasing the number of breeding pairs from about 20 to 150 pairs in the period 2007–2015 [7]. Nowadays, the population of rollers in Serbia is estimated to about 250–340 breeding pairs [11] and is highly dependent on the availability of artificial nest boxes, as the lack of nesting cavities seems to be the main limiting resource for this species in the Pannonian plain [7,8,12].

Although the number of breeding pairs of rollers in Serbia have recovered today, decline in the number of its breeding pairs in the 1990s [13–15] could have impacted the genetic variability of this population. Populations of smaller size are at risk of losing genetic variation through random drift and inbreeding depression, which can affect their long-term evolutionary potential and ability to respond to novel and changing environmental conditions [16]. Genetic depletion and higher inbreeding rates have been suggested as the leading causes of population decline in the roller population in Austria [17], which was confirmed by the observed loss of microsatellite diversity and nearly 90% decrease in effective population size between 2005 and 2015 [18]. Although the roller population in Serbia is nowadays demographically stable, genetic diversity estimation and genetic monitoring are needed to complement current conservation efforts, especially since the effects of demographic bottlenecks may persist for many generations [19] and depends on the region of origin of founder immigrants. In recovering populations, genetic diversity usually increases over time, which is related to a higher proportion of immigrating individuals. Immigration due to increased availability of nest boxes is thought to increase the rate of genetic recovery in populations following a bottleneck, but the underlying mechanisms are also influenced by life history and social and reproductive structures. Rollers have a tendency for natal philopatry and often breed near their natal sites and breeding dispersal may be smaller than natal dispersal [20]. Philopatric behavior may lead to the formation of discrete populations and suggest strong genetic structure, resulting in lower gene flow and potentially higher inbreeding. Furthermore, artificial increases in population density may even influence reproductive strategies (e.g., promoting extra-pair paternity, polygyny, and brood parasitism) and threaten long-term population viability [21].

The importance of conducting comparative studies on different populations of rollers in Europe has already been emphasized, as local populations may be affected by different limiting factors and therefore conservation measures may differ across species breeding range [6,22]. The roller population from Serbia is one of the most rapidly recovering and therefore provides a good example for assessing its genetic diversity following conservation efforts, especially since information on the genetic diversity of rollers across its range is generally limited [18,23]. In the current study, we analyzed 10 microsatellites loci in order to examine genetic diversity, estimate the level of inbreeding and effective population size in the recovered roller population from Serbia. In addition, all previously available mitochondrial DNA control region sequences were analyzed together with our newly generated sequences from Serbia to complement an earlier phylogeographic scenario of rollers in Eurasian context and to analyze the demographic history of this species.

## Materials and methods

### Sampling and DNA extraction

**Fig 1:**
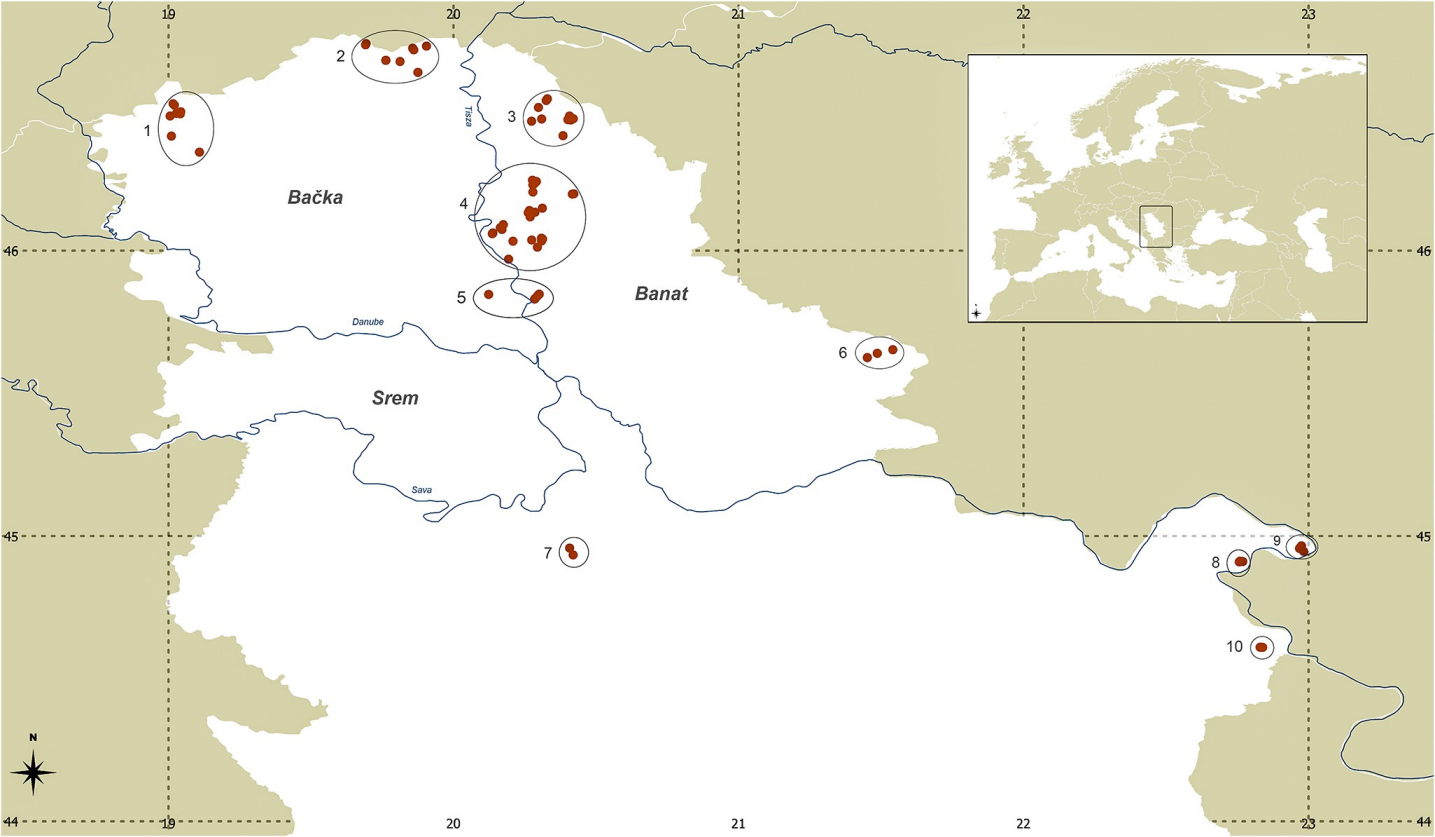
Map showing the sampling locations of the European roller individuals. Numbers correspond to ringing locations: 1. Sombor; 2. Subotica; 3. Mokrin; 4. Novi Bečej 5. Zrenjanin; 6. Vršac; 7. Barajevo; 8. Rtkovo; 9. Velesnica; 10. Srbovo.

Feather samples from 224 European rollers were collected at different localities in Serbia using a non-invasive method during bird ringing (Fig 1) from 2015 to 2020. Sampling was conducted as part of regular ringing of young birds, therefore the data set may also include close relatives from identical nests that were ringed shortly after hatching. The samples were collected by bird ringers—collaborators of the Centre for Animal Marking (Natural History Museum, Belgrade, Serbia), which has a license for bird ringing (353-01-2503/2015-17; 353-01-1432/2017-04; 353-01-4/2019-04; 353-01-8/2020-04) received from the Ministry of the Environmental Protection of the Republic of Serbia, which is responsible for handling the permits of ringing of strictly protected species. The samples were taken outside protected areas or inside protected areas with the permission of the protected area managers (as regulated by the above mention bird ringing permits). As only non-invasive feather sampling was carried out, no additional permits were required. Feathers were preserved in absolute ethanol, and we used the umbilicus (superior and/or interior), according to the method described by Horváth et al. [24]. Total genomic DNA was extracted using the innuPREP DNA Mini Kit (Analytik Jena, Germany) according to the manufacturer’s recommendations.

### Microsatellites genotyping

A total of ten microsatellite loci [25] were analyzed in two independent multiplex Polymerase Chain Reactions (PCR) using a commercial Type-it Microsatellite PCR kit (QIAGEN, Hilden, Germany). The first multiplex set included following loci: TG03-098, TG04-012, HvoB1, TG02-078, TG04-061, while the second set included microsatellite loci: TG01-040, TG03-002, TG08-24, TG05-053, SAP47. PCRs were conducted in a total volume of 12.5 μL per reaction, consisting of 6.25 μL Type-it Multiplex PCR Master Mix, 1.25 μL of 5X Q-solution, 10 μM of each primer, and 5 ng of genomic DNA. The PCR amplification conditions were as follows: an initial denaturation step at 95°C for 5 min, followed by 30 cycles of amplification, with each cycle comprising a denaturation step at 94°C for 30 s, annealing at 57°C for set 1 and 60°C for set 2 for 45 s, extension at 72°C for 30 s, and a final extension at 60°C for 10 min. Fragment analysis was conducted using capillary electrophoresis on an Applied Biosystems ABI3730XL DNA Analyzer (Applied Biosystems, Massachusetts, United States). Prior to fragment analysis, all samples were combined with Hi-Di formamide and GeneScan^™^ 500 LIZ^™^ dye size standard (Applied Biosystems, Massachusetts, United States). The genotyping was performed using GeneMarker software (Softgenetics, USA).

### Mitochondrial DNA control region sequencing

A partial fragment of the mitochondrial DNA (mtDNA) control region was amplified using *cogar7+* and *cogar5-* primers developed by Nebel et al. [18], yielding a 530 bp long amplicon. Approximately 50 ng of genomic DNA was amplified by PCR in a total volume of 25 μl containing 0.2 mM dNTP, 0.5 μM each of forward and reverse primers and 1U of DreamTaq polymerase (Thermo Fisher Scientific, Massachusetts, United States). PCR amplification conditions were as follows: an initial step of denaturation at 95°C for 5 min, followed by 30 cycles of amplification—each cycle being 95°C for 30s, 56°C for 30s and 72°C for 60 s—and a final extension step at 72°C for 7 min.

The PCR products were purified following the ExoSAP protocol, whereas DNA sequencing was performed by capillary electrophoresis on an ABI 3730xl Genetic Analyzer (Applied Biosystems, Massachusetts, United States). After inspection of the chromatograms, all nucleotide sequences of good quality were aligned using the ClustalW algorithm implemented in BioEdit 7.0.9.0 [26] while final adjustments were made by eye.

### Microsatellites data analyses

The presence of null alleles, stuttering and large allele dropouts were tested using PopGenReport [27] and Micro-Checker 2.2.3. [28]. Genetic diversity parameters, such as the number of alleles (N_A_), expected (H_E_) and observed heterozygosity (H_O_), and coefficient of inbreeding (F_IS_), were calculated using Genetix. 4.05 [29] while the individual heterozygosity was estimated using the R function Genhet. Deviation from Hardy-Weinberg equilibrium (HWE) was determined using Genepop 4.7.5. [30], with adjustment of significance following Bonferroni correction. Genetic relationships between all individuals were tested by relatedness analyses as implemented in ML-RELATE software [31]. Genetic variation among populations and within the population was evaluated using analysis of molecular variance (AMOVA) conducted in Arlequin [32].

To assess the genetic structure, a Bayesian model-based clustering approach was used as implemented in STRUCTURE 2.3.4 [33]. Initially, STRUCTURE analysis was performed on the entire dataset (which included 10 loci with and without locprior (population were defined based on ringing areas as Sombor, Subotica, Morkin, Novi Bečej, Zrenjanin, Vršac, Barajevo, Rtkovo, Velesnica, and Srbovo, see also Fig 1)). Additional STRUCTURE analysis was conducted excluding the locus TG053-053 (with and without "locprior" option). All STRUCTURE analyses were performed with 500,000 MCMC replicates, a burn-in of 200,000, and correlated allele frequencies, and with 20 independent runs for each value of K ranging from 1 to 10. The STRUCTURE results were summarized using the Clumpak [34], and the best K value was determined using Structure Harvester [35]. Model-free detection of genetic structuring was performed using the Discriminant Analysis of Principal Components (DAPC) in adegenet R package [36]. Function *find*.*clusters* was initially employed to ascertain the optimal number of clusters by evaluating the lowest value of the Bayesian Information Criterion (BIC), considering the pre-detected groups (the same a priori groups employed in the STRUCTURE analysis). Presence of spatial patterns in genetic structuring was tested in MEMGENE [37] R package. Genetic individual divergence across a landscape was visualized with Alleles in space [38] using the Genetic Landscape Shape interpolation using a distance weighting parameter (α) of 1 with grid settings of 50 × 50, and the latter was also used for analysis of correlation between geographic and genetic distances.

In order to assess the relative gene flow between groups, we utilized divMigrate [39] analysis in R package diveRsity [40], employing Gst, Nm, and D methods to evaluate asymmetries in gene flow.

Effective population size was calculated using the software LDNE [41] based on linkage disequilibrium method, for whole data set and for genetic clusters suggested by genetic structuring analyses, by using different threshold values of 0.05, 0.02, and 0.01. To estimate past changes in effective population size using microsatellite loci, we used VarEff v 1.2 R package [42]. This method is based on coalescent theory and uses approximate likelihoods in a Monte Carlo Markov chain approach. The analysis was performed using a two-phase mutation model (TPM) with proportion for multi-step mutations of c = 0.15, and a mutation rate prior of 10^−2^ [43,44], assuming a generation time (G) of 3.79 years for the European roller [45].

Signature of a genetic bottleneck was tested using the program BOTTLENECK [46], by applying three different mutation models: IAM—Infinite Allele Model, SMM—Stepwise Mutation Model and TPM—Two-Phase Model. Moreover, the software calculates the standard deviation (SD) for the expected heterozygosity balance. This computation subsequently derives the standardized deviation (DH/sd = He-Heq/sd) for each locus. A positive DH/sd indicates increase in heterozygosity, whereas a negative value indicates a deficit in heterozygosity. To identify allele losses that may serve as indicators of recent population bottleneck events, the Garza-Williamson (G-W) statistics [47] has been calculated in Arlequin.

### Mitochondrial DNA data analyses

The number of polymorphic sites, haplotype diversity (Hd), nucleotide diversity (π) and mean number of pairwise differences (k) were calculated using DnaSP v5.10 software [48]. To obtain a broader phylogeographic scenario of this species, all sequences obtained in this study are analyzed together with those previously published by Nebel et al. [18]. For each of the downloaded haplotypes, the exact number of individuals was taken from the original reference, resulting in final combined dataset containing 329 sequences. Phylogenetic relationships among haplotypes were analyzed by constructing a median-joining (MJ) network [49] using PopART (available at http://popart.otago.ac.nz). Bayesian phylogenetic tree was constructed using MrBayes [50] via CIPRES Science Gateway [51]. Four Markov chains were run simultaneously for 50 million generations, with trees sampling every 100 generations. The optimal substitution model was set as Hasegawa-Kishino-Yano with invariable positions and gamma distribution (HKY+G+I) as suggested by MEGA X [52]. The posterior distribution of all parameters was examined using Tracer v1.7. [53] and visualization of the Bayesian tree was done with FigTree v1.3.1 [54]. The tree was rooted using the Abyssinian roller (*Coracias abyssinicus*) sequence (GenBank accession number OP380491) as out-group.

To account for the geographical location of individuals, spatial clustering of individuals based on mtDNA control region sequences were performed in BAPS 6.0 [55]. Based on the posterior probabilities and likelihood values, we determined that the most likely number of clusters was between 3 and 5, so the final analysis was performed with two independent runs for K = 2 to 5, with 10 replicates. To determine the most likely ancestral geographic location the Bayesian approach to phylogeographic clustering (BPEC) package [56] was used within the R statistical software [57]. BPEC analyses were conducted using a two-dimensional dataset (geographic coordinates), and two MCMC chains were run for 100 million iterations. Parameters of nucleotide diversity were calculated for each of the defined groups using DnaSP as described above. Arlequin was used to estimate genetic differentiation among groups by calculating Φ_ST_ values and to perform AMOVA analysis. Mismatch distribution analysis [58,59] based on the pairwise differences was performed using Arlequin to test for deviations from the sudden expansion model. Tests of neutrality, Tajima’s D [60] and Fu’s Fs [61], were also computed using Arlequin, while DnaSP program was used to run F* and D* tests [62].

Changes in the effective population size (Ne) over time for each defined haplogroup were evaluated with the coalescent-based method Bayesian Skyline Plot [63], using BEAST v1.7 [64] via the CIPRES portal, and visualized in Tracer v1.7. Three independent runs were performed, each with a 100 million generations, with sampling every 1000 generations. The strict molecular clock was used, with mutation rate of 0.00135 substitution per lineage, as estimated in mtDNA control region sequences in white wagtail (*Motacilla alba*) [65].

## Results

### Microsatellites analyses

All ten microsatellite loci were successfully amplified in 224 rollers individuals. However, a significant presence of null alleles was detected in locus TG05-053 and it was not included in further analyses. A total number of alleles in data set was 66 (without locus TG05-053), varied from 4 in locus HvoB1 to 10 in case of locus TG04-061, resulting in a mean of 7.33 alleles per locus. The observed heterozygosity (0.392) was significantly lower than expected (0.643) heterozygosity, with an overall F_IS_ value of 0.393 (S1 Table). After Bonferroni correction, 6 of 9 loci deviated from HWE, which may indicate a heterozygosity deficit. This observation is supported by the fact that four of the nine loci showed a significant level of inbreeding, with values of the inbreeding coefficient (F_IS_) exceeding 0.5. Furthermore, relatedness analysis also indicated a high level of related individuals among the rollers from Serbia.

**Fig 2:**
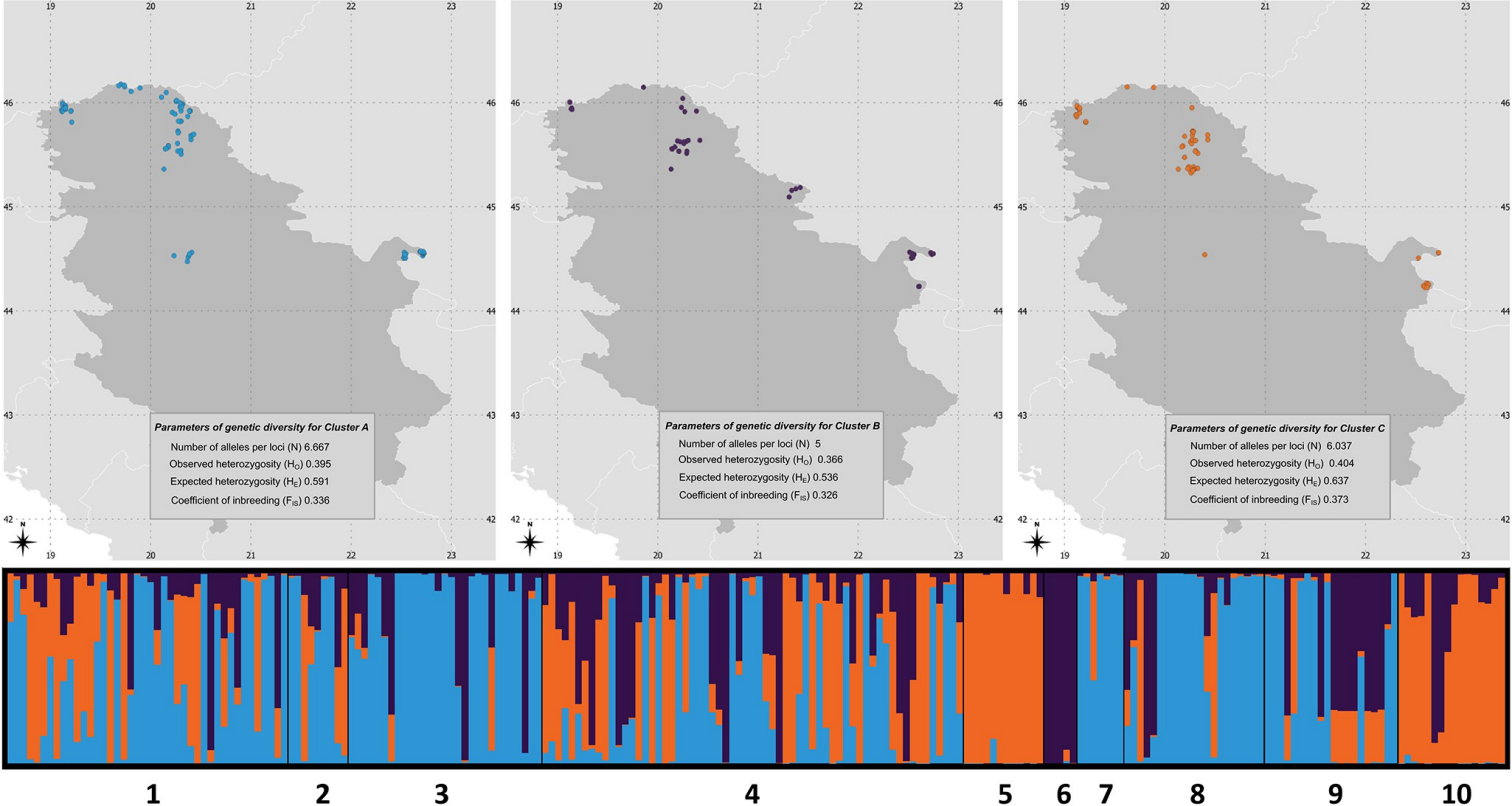
Barplot of genetic clusters obtained from STRUCTURE with maps corresponding to identified clusters A, B, and C, arranged from left to right. Within each map is a box with the genetic diversity parameters for the cluster it represents. Numbers of populations corresponds to rollers ringing locations as shown in Fig 1. The sampling points of each individual are jittered around the coordinates of the nest boxes for the spatial analyses.

Genetic clustering in STRUCTURE and DAPC yielded similar results, suggesting the presence of three main genetic groups named A, B and C (Figs 2 and S1), although lacking of clear spatial pattern (see also S2 Fig). However, individuals from Srbovo, a village in the municipality of Negotin (14 individuals; Fig 1, locality number 10) belonging to group C, were clearly separated from other individuals, as were individuals from Barajevo (6 individuals; Fig 1, locality number 7) belonging to group A, and individuals from Vršac (5 individuals; Fig 1, locality number 6) belonging to cluster B, which was further confirmed by the high genetic distances peaks in the landscape interpolation analyses (S3A Fig). Differentiation between detected clusters was low (average F_ST_ = 0.117) with only 12% of variation due to differences among groups (S2 and S3 Tables). Gene flow analyses revealed higher connectivity between cluster A and B, whereas gene flow between cluster C and the other two clusters was low. The nonsignificant r value (r = -0.018) obtained using the Mantel test indicated the absence of a correlation between geographic and genetic distances (S3B Fig).

The highest values of genetic diversity were observed in cluster C (H_E_ = 0.637; H_O_ = 0.404), while cluster B showed the lowest values (H_E_ = 0.536; H_O_ = 0.366) (Fig 2, S4 Table). Clusters A and C exhibited approximately similar average allele numbers per locus, whereas this value was lower for cluster B.

The estimated effective population size in the entire data set was highest when a threshold of 0.02 allele frequencies was applied (S5 Table). When considering each cluster separately, the effective population size was similar in clusters B and C, approximately 100 and 150 individuals, respectively, regardless of the allele frequency threshold applied. Bottleneck analysis did not reveal evidence of heterozygote excess for any of the tests applied (standardized differences and Wilcoxon tests) under I.A.M., T.P.M., and S.M.M. models, nevertheless, in cluster C, Wilcoxon test shown significant heterozygosity deficiency (p < 0.005) under SMM model. Negative DH/sd was observed in 6 out of 9 loci throughout the entire population, consistently across almost all cases, indicating a significant heterozygote deficit in those loci. The values of the Garza-Williamsons statistic indicate that the roller population in Serbia has not experienced a significant loss of alleles. This observation is consistent with the results of the Bottleneck analysis. Estimates of changes of effective population size over the past indicated a decline over the last 100 generations (S4 Fig).

### Mitochondrial DNA analyses

**Fig 3:**
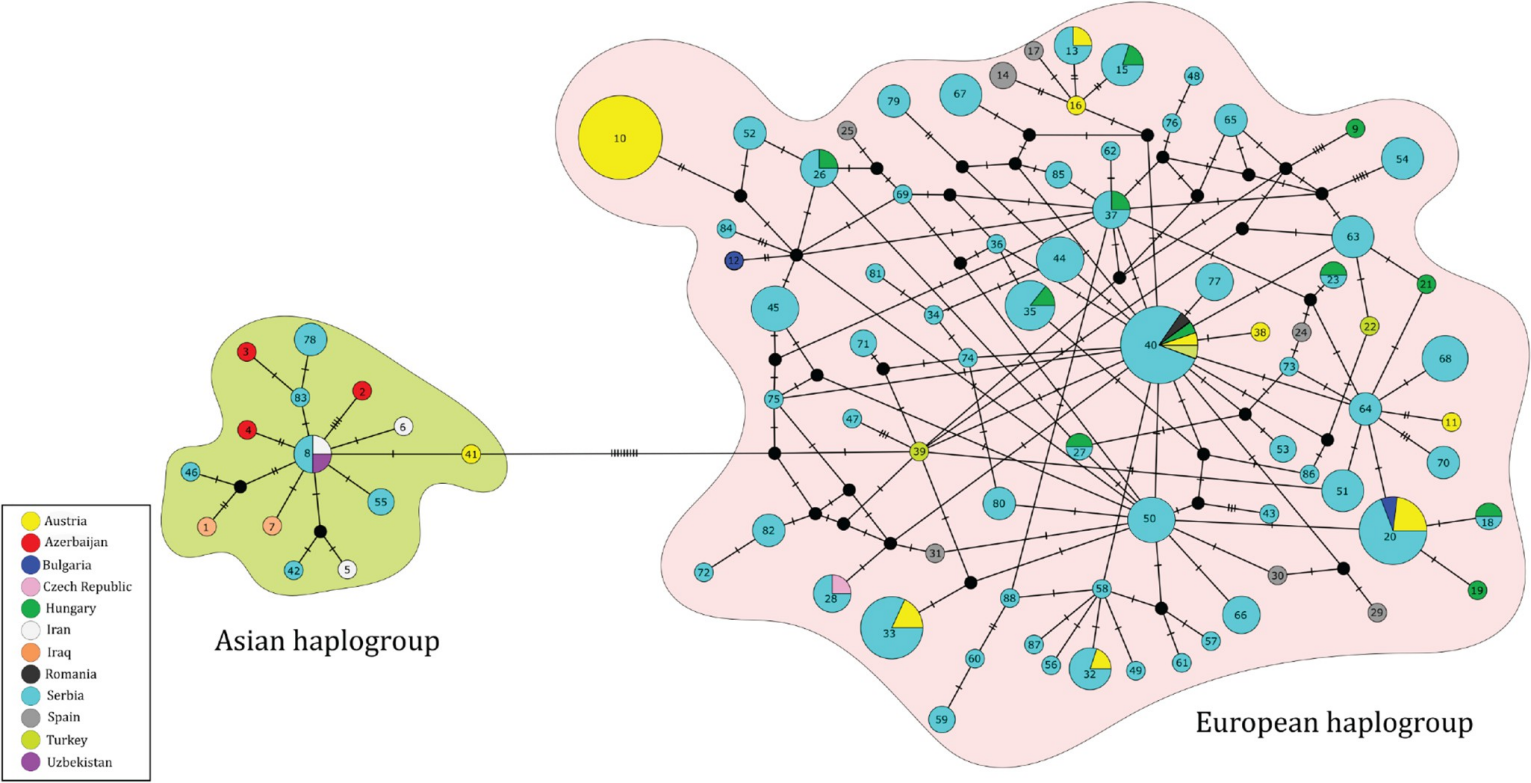
Median-joining network of the mtDNA control region haplotypes of European roller. Circles are colored according to geographical distribution of haplotypes. Circle sizes are proportional to haplotype frequencies. Black dots represent median vectors. Haplotype designations are as in S6 Table, with additional information on haplotype frequency and sampling location.

In 224 European rollers from Serbia, 61 haplotypes of the nucleotide sequences of the mtDNA control region were detected based on 53 polymorphic sites, 46 of which were parsimony informative. Of the 61 haplotypes discovered in rollers from Serbia, 47 had not been discovered in previous studies, 14 of which were unique. Most of the newly detected haplotypes occurred at low frequencies, while the most common haplotype (Cogar40) had already been detected in roller populations from Austria, Hungary, and Romania. Among the haplotypes unique to the population from Serbia, Cogar44, Cogar45 and Cogar50 were the most frequent (Fig 3).

The combined data set of sequences from this study, analyzed together with the publicly available sequences, revealed 88 haplotypes based on the alignment of 329 sequences with a final length of 440 bp (considering sites with gaps). Although the length of our submitted sequences is 469 bp, all analyses were based on the shorter alignment of 440 bp, as we could not achieve the full amplicon length of 469 bp in four samples. As indicated by Bayesian phylogenetic tree and median-joining network two major haplogroups can be distinguished: European haplogroup and Asian haplogroup (Figs 3 and S5). The observed grouping was further supported by the AMOVA analysis, which showed that 71% of the variation was due to the differences between the groups, with an Φ_ST_ value of 0.712 (p < 0.01). In general, higher molecular diversity indices were observed in the European group compared to Asian haplogroup (Table 1). However, the highest values of molecular diversity were found when only the roller population from Serbia was considered (Table 1).

**Table 1:** Molecular diversity indices, mismatch distribution analysis, and neutrality test for European (EU) and Asian (ASN) haplogroups and total dataset based on the control region mtDNA sequences in *Coracias garrulus*.

Molecular diversity	Serbia	EU	ASN	Total
Number of sequences (n)	224	304	25	329
Number of haplotypes (h)	61	74	14	88
Number of polymorphic sites (S)	53	52	20	62
Haplotype diversity (Hd)	0.987	0.958	0.92	0.964
Nucleotide diversity (π)	0.012	0.01	0.006	0.013
Average number of pairwise differences (k)	5.423	4.516	2.68	5.927
**Mismatch distribution and neutrality test**				
Sum of squared deviations (SSD)	0.007	0.002	0.001	0.006
Harpending’s raggedness index	0.017	0.016	0.031	0.013
Tajima’s D	-1.18	-1.339	-1.785*	-1.162
Fu’s Fs	-32.38**	-25.146**	-7.399**	-24.651**
Fu and Li’s F*	-0.449	-1.027	-2.409	-0.998
Fu and Li’s D*	0.28	-0.444	-2.18	-0.536

*p <0.05

**p<0.01.

The European haplogroup contains individuals mostly occurring in Austria, Spain, Hungary and Serbia, while the birds belonging to Asian haplogroup can be found in Iran, Azerbaijan, Iraq and Uzbekistan, but also in Serbia. The most common haplotype within the European haplogroup (Cogar40) occurred in rollers from Austria, Hungary, Romania, Turkey, and Serbia. However, no clear spatial signal was observed when considering only European haplogroup (S6 Fig). The most common haplotype inside the Asian haplogroup, haplotype Cogar8 was detected in rollers from Uzbekistan and Iran, but also in rollers from Serbia and it is a shared haplotype present in both subspecies (*C*. *garrulus garrulus* and *C*. *garrulus semenowi*). Within the Asian clade only two unique haplotypes are distributed in rollers from Vojvodina province (Cogar46 and Cogar83). In addition to all the above, the haplotype Cogar78 detected in individuals from Serbia clustered into the Asian haplogroup.

The mismatch distribution did not deviate statistically significantly from the values expected under a sudden expansion model in either the overall dataset or in either haplogroup (Table 1, S7 Fig). Neutrality test values were negative, but only Fu’s Fs value showed significant deviation from expected which can indicate recent demographic expansion. The Bayesian skyline plot indicated rather stable changes in effective population size over time when performed separately for each haplogroup, while decline was observed when considering the full dataset starting around 25 Kya (S8 Fig).

## Discussion

### Moderate genetic diversity and shallow genetic structuring in rollers from Serbia

Moderate genetic diversity in rollers from Serbia was detected with significant lower observed than expected heterozygosity, which is at a similar level as observed in previous studies in roller populations in Europe [18,23]. In the roller population from Spain, almost the same value of observed heterozygosity was found (0.357), based on the variability of 10 microsatellite loci [23], whereas the heterozygosity observed in the roller population from Austria [18] with the same set of microsatellite loci as used in our analysis is higher (although not fully comparable to our results because most of their samples are from 2005 and 2007). However, the number of alleles per locus detected in our study remains relatively high which indicates a continuous increase in genetic variability. Contrary to the roller population from Austria, where a decline resulting in the loss of rare alleles has been observed [18], our results does not indicate evidence of current genetic erosion in the roller population from Serbia.

Genetic structuring results revealed the existence of three large clusters with shallow divergence and no clear spatial pattern. This pattern of genetic structure is common in migratory birds, as they are not limited by the existence of landscape barriers in the way terrestrial species are. In birds, the level of gene flow is shaped by two opposing natural processes: their ability to migrate and disperse during flight routes, which significantly contributes to gene flow, and, on the other hand, their philopatric nature which mitigates this effect [66]. Population genetic structuring is influenced by the spatial distribution of populations outside of the breeding areas. Contrary to weak migratory connectivity in western rollers populations [67], stronger connectivity is observed in eastern populations most likely as consequence of continental configuration enabling these population to maintain their longitudinal structure [68]. Therefore, the genetic structuring observed during the breeding season may be influenced by the spatial distribution of wintering populations and their migratory connectivity, however further studies are needed to clarify these effects on the observed genetic structuring patterns in rollers from Serbia. In addition, the installation of artificial nest boxes on favorable roller habitats has created an avenue for immigrants from different European populations, and it remains uncertain to what extent immigration from other neighboring populations influences observed genetic structure. Philopatry, noticed in several roller populations in Europe [20,69], can also have a major impact on the genetic structure of populations. It’s noteworthy to mention that environmental factors within roller habitats not only influence the formation of fine-scale distribution patterns but can also impact the finer-scale genetic structure, a characteristic often observed in migratory bird species [70]. In addition to all of the above, the European Roller has shown strong preferences for specific habitat types [5,71–74].

Although a clear spatial pattern was not evident, a significant genetic differentiation was noticeable among the majority of individuals originating from a specific ringing locality within the Negotin region (Srbovo, Fig 1, locality number 10), setting them apart from the broader population. This region is one of the localities where rollers still mostly inhabit natural holes, distinguished by its historical habitat and abundant natural breeding grounds, with a particular abundance of old trees [75]. Genetic differentiation of this ringing region was also supported by our gene-flow analyses, which showed that there is no gene flow between cluster C (consisting of individuals from Srbovo) and the populations of the other two clusters. It is important to note that this location is located on the border near Romania, where populations with thousands of breeding pairs exist. However, a higher gene flow was observed between individuals from the other two clusters (A and B), indicating greater connectivity between them, possibly due to the fact that rollers in these ringing areas inhabit artificial nesting boxes compared to natural nesting cavities in trees [12].

Previous research has suggested that rollers exhibit philopatry behavior and a tendency for colonial formation [76]. Installation of these boxes could significantly influence population structure, colony formation, and potentially accelerate philopatry. This trend is observed in the roller population in Serbia as well, where monitoring of established populations has revealed an annual occurrence of new breeding pairs within a range of 5–15 km southeast beyond the previously known breeding area [7], which strongly suggests the species’ tendency to exhibit site fidelity and a preference for returning to favored breeding localities. In addition, field observation indicates a higher proportion of rollers from Serbia inhabiting artificial breeding boxes compared to natural breeding holes in trees, further reinforcing the idea that the installation of boxes can shape population structure, foster colonies, and expedite potential philopatry. Philopatry has greater impact on small, isolated groups and additionally increases the possibility of crossing closely related individuals, reduces gene flow as well as genetic variability [77]. Furthermore, this may potentially result in heightened relatedness and inbreeding within the roller population. Indeed, increased inbreeding detected in our results is evident in the significantly low level of heterozygosity and elevated F_IS_ value, which also to some extent may be linked to the sampling of closely related individuals (although F_IS_ values did not differ significantly when first degree relatives were removed). The lowest F_IS_ value is observed in Srbovo, which is mentioned above as one of the localities where birds largely inhabit natural breeding places. Another locality with low F_IS_ value is Barajevo, which represents one of the most isolated ringing localities, with recently established monitoring and nesting box arrangements [78]. A high level of homozygosity, inbreeding and relatedness is a pattern that is expected in bird populations with philopatric nature. Besides that, roller population from Serbia/Vojvodina province experienced an increase in the number of individuals in the last two decades during intensive recovery and monitoring programs and a rise from a small number of individuals. Although Nebel et al. (2019) [18] asserted that the roller population from Serbia is stable, without evidence of a drastic decrease in recent history, it is important to state that in their dataset all individuals from Serbia are sampled from 2015 at the time when recovery program was already in progress.

### Artificial nesting boxes prompt roller populations from Serbia

Installing of artificial nesting/breeding boxes has a significant positive impact on the roller population in Serbia. Historical data from ringing reports during the 1990s indicated a low number of recorded breeding pairs, especially in northern Serbia [9]. However, since the monitoring started in 2003 (initially in the region of Subotica and expanded on the other locations in 2007), the number of breeding pairs has consistently doubled each year [7]. Presently, we can state that the roller population in Serbia is relatively stable. The preference of birds for artificial nesting boxes suggests that their installation directly contributed to the population’s rapid increase in a short period of time. From the abundance data, it can be inferred that the population faced a risk of extinction at the beginning of the century and underwent a bottleneck. The absence of a current bottleneck in our analysis indicates that the roller population in Serbia is now stabilizing. The only case of heterozygous deficiency was observed in cluster C, which supports the hypothesis of Srbovo region’s separation from the rest of the rollers from Serbia. This finding also indicates that this population still retains traces of a bottleneck event and may represents remnants of the older roller populations.

The analysis of the effective population size suggests a close correspondence between the Ne value obtained and the current number of breeding pairs recorded through monitoring and ringing. Based on the recommendations of conservation biologists, the 50/500 rule has been applied for years as a guideline when assessing the effective size of a population [79]. However, recent studies have even proposed a reconsideration of the 50/500 rule, suggesting that doubling this value might be essential to ensure a population’s sustainable long-term evolutionary potential [80,81]. Despite observing a notable increase in the population size over the past few years, as confirmed by analyses of historical changes in effective size, the roller population in Serbia has not yet reached these ideal values. Although the population is currently on the rise, it remains imperative to implement conservation measures and establish regular monitoring protocols.

A positive impact of the arrangement of artificial nest boxes on avian populations has been consistently observed throughout Europe. The strategic placement of nest boxes near the most abundant prey reduced the effort required to find and transport food for the offspring. This increased accessibility of food contributed to increased fecundity, leading to the successful recovery of the endangered bird species [82–84]. This was also found in several roller populations in Europe (Italy [71], France [85], Spain [86]), and in most cases the artificial nests had significantly higher occupancy rates compared to natural nests, which is in correlation with the results recorded during the ringing of the roller population in Serbia. One of the reasons for this could be that artificial nests are more noticeable compared to nesting cavities in old trees, which are usually better hidden [87]. While the installation of wooden nest boxes has undoubtedly yielded a highly positive effect on the population, it is essential to acknowledge that such placement can also influence the population dynamics and impact the behavior and social strategies of the species. Previous research has revealed that nest selection preferences vary among different roller populations [8,22,71], emerging as a pivotal factor significantly impacting breeding and nesting success. Therefore, when situating artificial nest boxes, it is very important to consider not only the attributes of the habitats themselves but also the preferences of the local roller population, which could be region-specific. Furthermore, it is well-established that species nesting within cavities and displaying a tendency for colony formation are also prone to adopting alternative reproductive strategies [88–90], such as extra-pair paternity (e.g. *Riparia riparia* [91], *Parus caeruleus* [92]) and conspecific brood parasitism (e.g. *Passerculus sandwichensis* [93]). A comprehensive study of the roller population from Spain revealed through microsatellite loci analysis that the species does not strictly adhere to monogamy but rather employs alternative reproductive tactics involving additional pairs of both males and females [23]. While the precise factors driving these avian adaptations to alternative strategies remain partially understood, their discernible impact on population dynamics is undeniable [88,89]. Several hypotheses regarding the origins of conspecific brood parasitism suggest that females might adopt this strategy during unfavorable environmental conditions, such as limited food resources [88]. Consequently, given that the installation of artificial nesting structures directly contributes to a rapid increase in population numbers, it is plausible that these structures could potentially foster the adoption of such alternative reproductive strategies. Alongside the ongoing monitoring and establishment of new nesting houses, regular surveillance of adult individuals and the banding of young individuals remain crucial. Additionally, concerted efforts should focus on restoring old, archaic habitats where feasible, and reintroducing the species to its natural environments. These measures collectively enhance the prospects of the species’ long-term recovery and conservation.

### Haplotypes of rollers from Serbia found in European and Asian haplogroups

By combining the sequences obtained from this study with those already available [18], a higher level of structuring within the European roller population was observed, as well as a less pronounced divergence between the European and Asian clades than previously considered. Our Bayesian tree and median-joining network has shown that samples from across Europe (contemporary and historical samples from museums) and those from Turkey were a part of the European group, while those from Azerbaijan, Iran, Iraq, and Uzbekistan were clustered in the Asian group. In contrast to the study of Nebel et al. [18], which assumed that the distribution of haplogroups could underpin the subspecies distinction, assuming that the two haplogroups are parapatric, our study revealed a number of haplotypes discovered in Serbia belonging to the Asian haplogroup. This may indicate an incomplete lineage sorting or rather a wider distribution of both subspecies in past. According to the data based on *Miocoracias* fossils from the early Miocene discovered in France, the rollers had a much wider distribution and large breeding distribution across Europe [94]. Indeed, the geographic distribution of haplotypes suggests that the rollers in northern Serbia (Vojvodina province) are more similar to those in western populations, such as Hungary and Austria, but in addition rollers from northern Serbia are also more alike to the historical population from Austria than the population found in Austria today. Comparing the results obtained from historical samples in Austrian museum collections with the results of this study, it is evident that some haplotypes previously considered unique are also detected in Serbia (e.g., Cogar13, Cogar28, and Cogar32). According to our results, the most likely ancestral roller haplotype originates from Turkey, which corresponds with a theory about a continual roller population throughout Asia and Africa. However, *C*. *garrulus* originally come from Africa [95], which is also supported by the phylogenetic tree clustering *C*. *garrulus* together with the African rollers *Coracias caudatus* and *Coracias abyssinicus*. Consistent with previous studies and traditional subspecies classification [1] *C*. *g*. *semenowi* occupied the middle east (Iraq, Iran, and Easter more) and our results also support that *C*. *g*. *semenowi* has a wider distribution and are moving further west [18], especially since some haplotypes previously detected only in samples from the Asian continent are found in rollers geographically sampled in Serbia. However, the relationship between the two subspecies is relatively complex and correlation between genetic pattern and geographic distribution should be interpreted with cautions [96].

Our Bayesian analysis of genetic structuring based on mtDNA sequences indicated the existence of four genetic clusters, three of which are detected among individuals sampled in Serbia, however, not with a consistent spatial pattern. The fact that individuals belonging to three clusters were discovered in the population from Serbia may indicate high genetic diversity and support the southern refugia theory [97,98] that the Balkans may have served as a refuge for European rollers during the Pleistocene glaciation and contributed to their genetic diversity [99]. Moreover, the observed distribution of clusters in our analysis correlates to some extent with the migratory routes of European rollers with a noticeable division into western and eastern routes [68]. This may indicate that migratory routes have played some role in shaping the distribution of genetic clusters and genetic structure. While Finch et al. [68] suggests that all individuals from Serbia could have migrated through what is referred to as the eastern route, our analysis of cluster distribution does not conclusively ascertain whether individuals in the northern and eastern regions of Serbia follow the same migratory routes or initiate their migration simultaneously.

The demographic expansion of the European roller started in recent history, as indicated by negative values of neutrality tests, which is further supported by the combination of high haplotype diversity and low nuclear diversity. The unimodal mismatch distribution also supports recent demographic expansion, although a small second peak can be noticed, which may indicate that the population underwent immediate expansion through allopatric speciation. Population size changes over time showed that the European haplogroup has oscillated over the past 100,000 years, from a drastic decline to a slight increase in more recent history. The effective population size of the Asian clade has been more constant throughout history. However, these demographic inferences should be considered with cautions as are based on the usage of mutation rate from a species not closely related to the European roller.

## Conclusions

The roller population in Serbia has recently experienced a remarkable recovery. In the last two decades alone, the number of breeding birds has increased almost 10 times. Our study has shown that the population of the roller from Serbia has moderate level of genetic diversity and there are no signs of recent bottleneck. No clear genetic structuring patterns are noticed and no signal of isolation by distance. Nevertheless, roller population from Serbia is still dependent on artificial nesting sites. Installation of artificial nest boxes has a double effect. On the one hand, it increases fecundity and fertility, which leads to an increase in breeding pairs. On the other hand, the placement of nest boxes also affects colony formation and increases philopatry, possibly contributing to the relatively high degree of inbreeding observed in the population. Therefore, it is critical to continue genetic monitoring of this population and to strategically place these nest boxes in new, favorable habitats, which would not only facilitate the formation of new pairs, but also reduce the possible increase of inbreeding and negative consequences of philopatric behavior.

## Supporting information

S1 Fig DAPC analysis scatterplot and BIC values for European roller (*Coracias garrulus*) from Serbia. (PDF)

S2 Fig Spatial genetic structure of European roller (*Coracias garrulus*) from Serbia as revealed by MEMEGENE. (PDF)

S3 Fig Genetic Landscape Shape interpolation and Mantel test results in European roller (*Coracias garrulus*) from Serbia. (PDF)

S4 Fig Estimates of effective population size (Ne) over the past of the European roller (*Coracias garrulus*) from Serbia based on the microsatellites data. (PDF)

S5 Fig Bayesian tree of mtDNA control region haplotype sequences of European roller (*Coracias garrulus*). (PDF)

S6 Fig Map showing the genetic clusters identified by (BAPS) and BPEC results based on mtDNA control region sequences in European rollers (*Coracias garrulus*). (PDF)

S7 Fig Mismatch distribution graphs for Serbia, European and Asian haplogroup of European roller (*Coracias garrulus*). (PDF)

S8 Fig Bayesian skyline plots of European roller (*Coracias garrulus*) for Serbia, European and Asian haplogroup. (PDF)

S1 Table Genetic variability parameters in European roller (*Coracias garrulus*) from Serbia based on the microsatellites data. (PDF)

S2 Table Results of AMOVA analysis for detected genetic clusters in European roller (*Coracias garrulus*) from Serbia based on the microsatellites data. (PDF)

S3 Table Pairwise F_ST_ values between detected genetic clusters in European roller (*Coracias garrulus*) from Serbia. (PDF)

S4 Table Genetic variability for detected genetic clusters in European roller (*Coracias garrulus*) from Serbia based on the microsatellites data. (PDF)

S5 Table Effective population size (Ne) in European roller (*Coracias garrulus*) from Serbia based on the microsatellites data. (PDF)

S6 Table List of mtDNA control region haplotypes of *Coracias garrulus*, with frequency and sampling locality for each haplotype. (PDF)
