# Supplementary material for: Genetic diversity of a recovering European roller (Coracias garrulus) population from Serbia
Source: PLoS One. 2024 Aug 8;19(8):e0308066. doi: 10.1371/journal.pone.0308066 (PMC11309509; doi:10.1371/journal.pone.0308066)
Supplement: S1 Fig — (PDF) [file pone.0308066.s001.pdf]

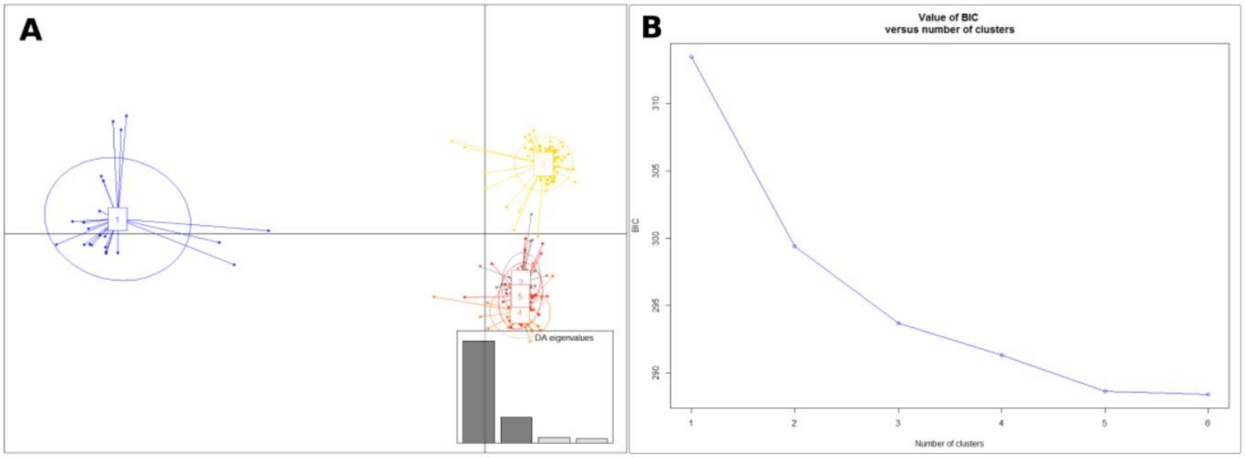

Figure S1. Scatterplots from discriminant analysis of principal components (DAPC) (A) and BIC values (B) in European roller (*Coracias garrulus*) from Serbia. Each ellipse represents a cluster, each dot represents an individual, and eclipses represent 95% confidence.
