# Supplementary material for: Genetic diversity of a recovering European roller (Coracias garrulus) population from Serbia
Source: PLoS One. 2024 Aug 8;19(8):e0308066. doi: 10.1371/journal.pone.0308066 (PMC11309509; doi:10.1371/journal.pone.0308066)
Supplement: S2 Fig — (PDF) [file pone.0308066.s002.pdf]

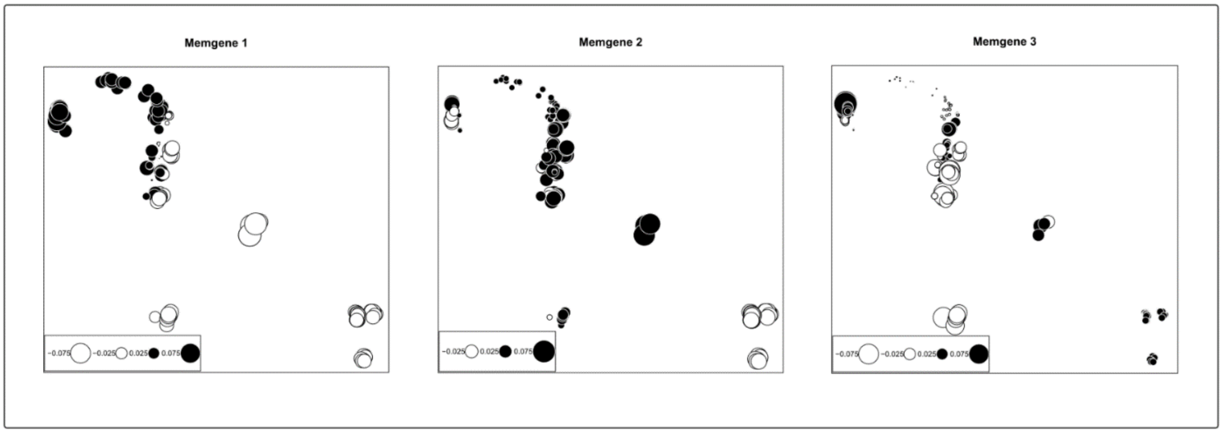

Figure S2. Visualizations of the spatial genetic structure of European roller (*Coracias garrulus*) from Serbia using MEMEGENE. Circles of a similar size and color indicate individuals with similar scores (large black and white circles describe opposite extremes on the MEMGENE axes). Results are shown for first three components named Memgene 1–3.
