# Supplementary material for: Genetic diversity of a recovering European roller (Coracias garrulus) population from Serbia
Source: PLoS One. 2024 Aug 8;19(8):e0308066. doi: 10.1371/journal.pone.0308066 (PMC11309509; doi:10.1371/journal.pone.0308066)
Supplement: S3 Fig — (PDF) [file pone.0308066.s003.pdf]

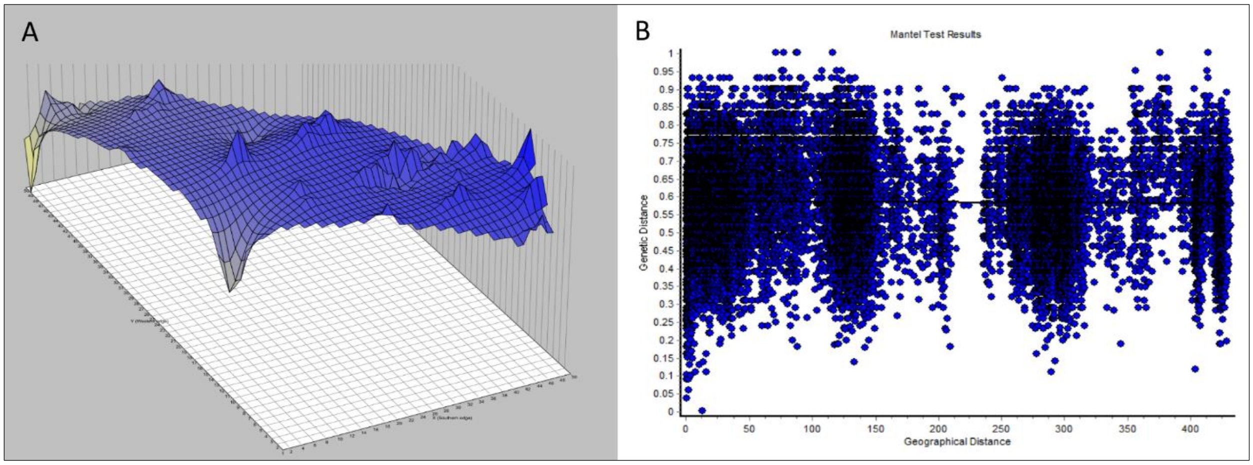

Figure S3. A. Genetic Landscape Shape interpolation in European roller (*Coracias garrulus*) from Serbia based on a 50 × 50 grid and a surface plot height proportional to genetic distances. **B**. Mantel test results for European roller (*Coracias garrulus*) population from Serbia.
