# Supplementary material for: Genetic diversity of a recovering European roller (Coracias garrulus) population from Serbia
Source: PLoS One. 2024 Aug 8;19(8):e0308066. doi: 10.1371/journal.pone.0308066 (PMC11309509; doi:10.1371/journal.pone.0308066)
Supplement: S4 Fig — (PDF) [file pone.0308066.s004.pdf]

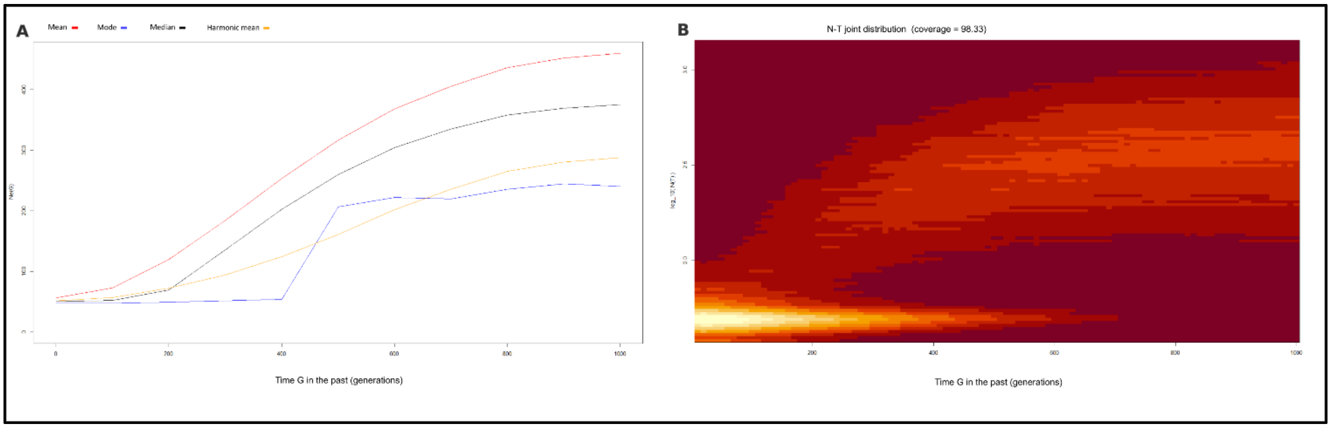

Figure S4. Estimates of effective population size (Ne) in the past of the European roller (*Coracias garrulus*) population from Serbia. **A**. Ne from today to the last 1000 generations. **B.** Posterior distribution of effective population size in the past during the last 1000 generations. The colored lines indicate the arithmetic mean of Ne (red), harmonic mean (orange), mode (blue), and median (black), respectively.
