# Supplementary material for: Genetic diversity of a recovering European roller (Coracias garrulus) population from Serbia
Source: PLoS One. 2024 Aug 8;19(8):e0308066. doi: 10.1371/journal.pone.0308066 (PMC11309509; doi:10.1371/journal.pone.0308066)
Supplement: S5 Fig — (PDF) [file pone.0308066.s005.pdf]

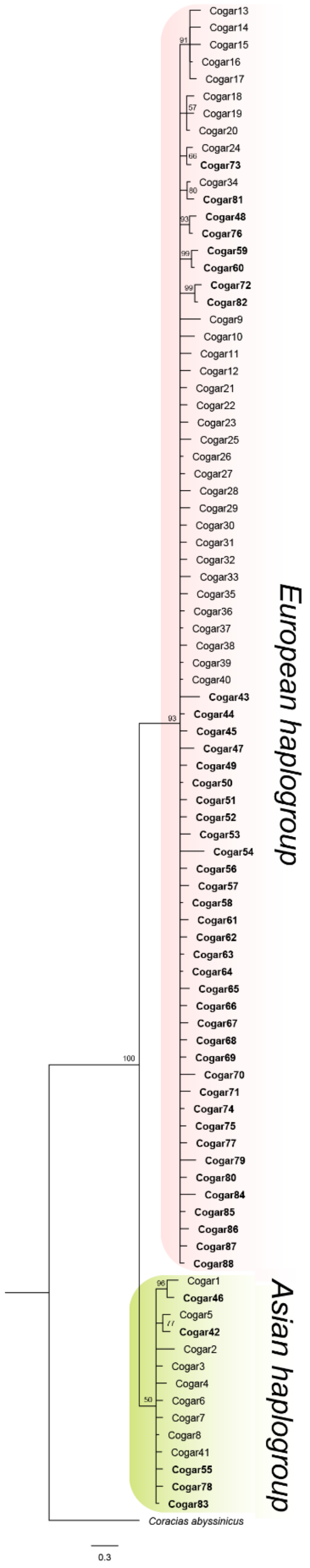

Figure S5. Bayesian tree based on haplotypes of the mtDNA control region of European roller (*Coracias garrulus*). *Coracias abyssinicu*s is used as the outgroup. Haplotypes newly identified in this study are highlighted in bold.
