# Supplementary material for: Genetic diversity of a recovering European roller (Coracias garrulus) population from Serbia
Source: PLoS One. 2024 Aug 8;19(8):e0308066. doi: 10.1371/journal.pone.0308066 (PMC11309509; doi:10.1371/journal.pone.0308066)
Supplement: S6 Fig — (PDF) [file pone.0308066.s006.pdf]

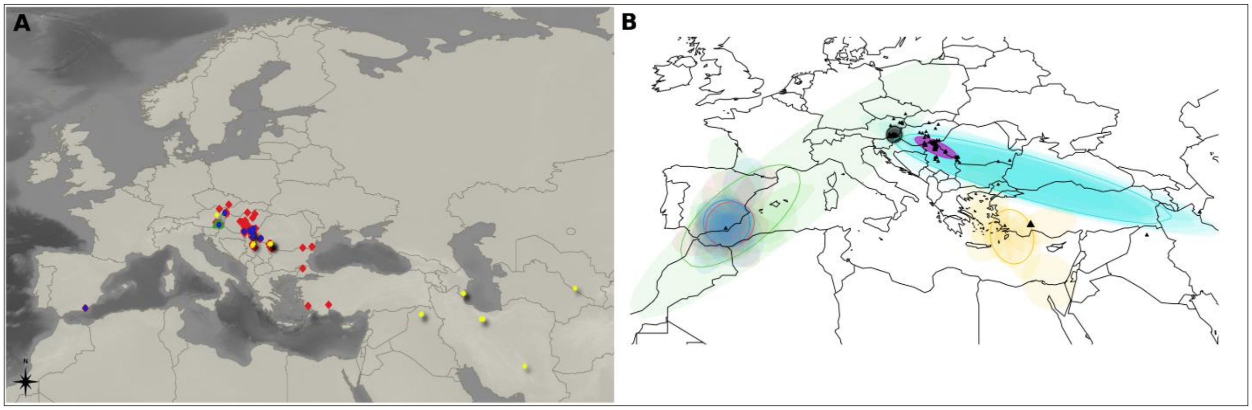

Figure S6. A. Map showing the four genetic clusters identified by Bayesian analysis of population structure (BAPS) in European rollers (*Coracias garrulus*). The colors and shapes represent the cluster to which the analyzed individuals belong based on BAPS assignment. **B.** Results of Bayesian phylogeographic and ecological clustering (BPEC). Each colored area indicates a different phylogeographic grouping, while biggest triangle represents the likely ancestral haplotype.
