# Supplementary material for: Genetic diversity of a recovering European roller (Coracias garrulus) population from Serbia
Source: PLoS One. 2024 Aug 8;19(8):e0308066. doi: 10.1371/journal.pone.0308066 (PMC11309509; doi:10.1371/journal.pone.0308066)
Supplement: S7 Fig — (PDF) [file pone.0308066.s007.pdf]

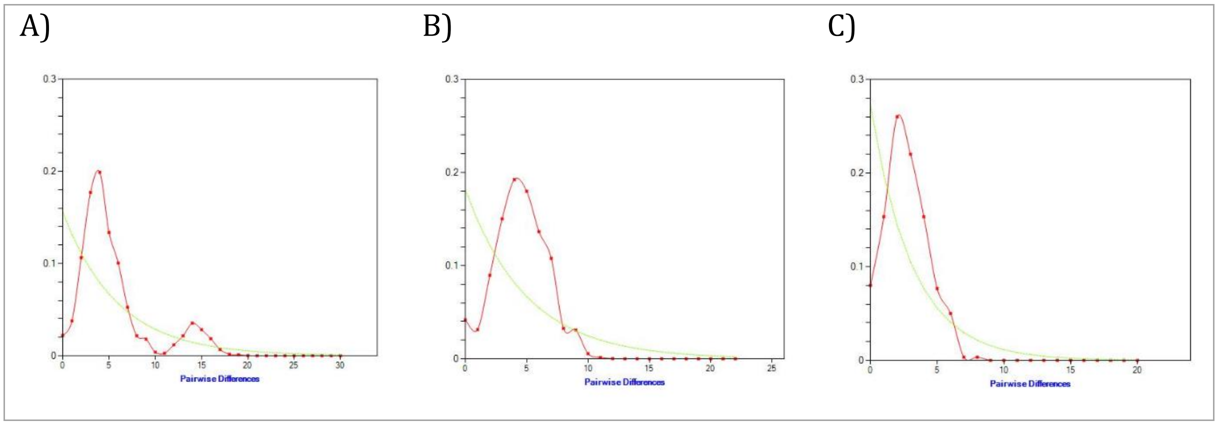

Figure S7. Mismatch distribution graph for European roller population (*Coracias garrulus*) from Serbia (**A**), European (**B**) and Asian (**C**) haplogroup. The x axis shows the number of pairwise differences, the y axis shows the frequency of the pairwise comparisons. The observed frequencies were represented by red dotted line. The frequency expected under the hypothesis of population expansion model was depicted by green line.
