# Supplementary material for: Genetic diversity of a recovering European roller (Coracias garrulus) population from Serbia
Source: PLoS One. 2024 Aug 8;19(8):e0308066. doi: 10.1371/journal.pone.0308066 (PMC11309509; doi:10.1371/journal.pone.0308066)
Supplement: S8 Fig — (PDF) [file pone.0308066.s008.pdf]

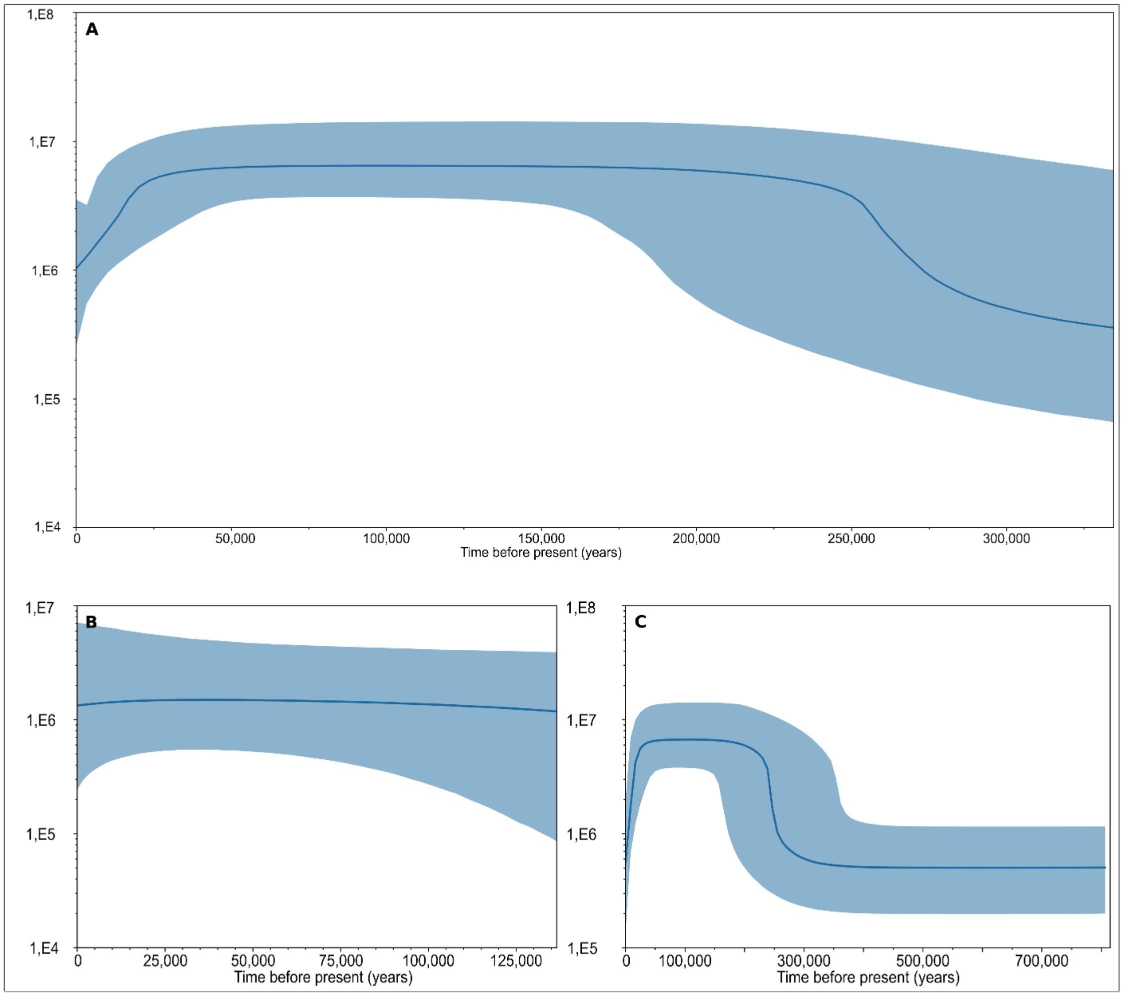

Figure S8. Bayesian skyline plots obtained from the mtDNA control region sequences of European roller (*Coracias garrulus*) for European haplogroup (A), Asian haplogroup (B), and Serbia (C). The x axis is in calendar years before present, and y-axsis represents changes in effective population size shown as the product of Ne and generation time. The thick solid line represents the mean effective population size Ne, while the blue solid area represents the 95% highest probability density intervals.
