# Supplementary material for: Genetic diversity of a recovering European roller (Coracias garrulus) population from Serbia
Source: PLoS One. 2024 Aug 8;19(8):e0308066. doi: 10.1371/journal.pone.0308066 (PMC11309509; doi:10.1371/journal.pone.0308066)
Supplement: S1 Table — (PDF) [file pone.0308066.s009.pdf]

Table S1 Genetic variability parameters in European rollers (*Coracias garrulus*) from Serbia.

Locus	N	Na	Ho	He	F_{IS}	GW
HvoB1	4	1.146	0.103	0.128	0.197	0.571
TG04-012	7	3.173	0.230	0.685	0.666	1.000
TG04-061	10	5.484	0.558	0.818	0.319	0.769
TG03-098	9	5.252	0.524	0.810	0.355	0.900
TG02-078	5	3.042	0.596	0.671	0.114	0.384
TG03-002	9	1.618	0.167	0.382	0.565	1.000
SAP47	5	3.891	0.664	0.743	0.109	0.555
TG08-24	8	4.433	0.347	0.774	0.553	0.888
TG01-040	9	4.483	0.339	0.777	0.565	1.000
**Mean**	**7.333**	**3.614**	**0.392**	**0.643**	**0.393**	**0.785**

N – number of alleles per locus; Na – effective number of alleles; He – expected heterozygosity; Ho – observed heterozygosity; F_{IS} – coefficient of inbreeding; GW - Garza-Williamson index.
