# Supplementary material for: Genetic diversity of a recovering European roller (Coracias garrulus) population from Serbia
Source: PLoS One. 2024 Aug 8;19(8):e0308066. doi: 10.1371/journal.pone.0308066 (PMC11309509; doi:10.1371/journal.pone.0308066)
Supplement: S2 Table — (PDF) [file pone.0308066.s010.pdf]

Table S2 Results of AMOVA analysis for European roller (*Coracias garrulus*) genetic clusters from Serbia.

Source of variation	*Percentage of variation*
Among populations	11.7
Among individuals within populations	30.18
Within individuals	58.11
F_{IS}	0.342
F_{ST}	0.117
F_{IT}	0.419
