# Supplementary material for: Genetic diversity of a recovering European roller (Coracias garrulus) population from Serbia
Source: PLoS One. 2024 Aug 8;19(8):e0308066. doi: 10.1371/journal.pone.0308066 (PMC11309509; doi:10.1371/journal.pone.0308066)
Supplement: S3 Table — (PDF) [file pone.0308066.s011.pdf]

Table S3 Pairwise F_{ST} values between A, B and C genetic clusters identified in European rollers (*Coracias garrulus*) from Serbia

	A	B	C
A	-		
B	0.156	-	
C	0.106	0.095	-
