# Supplementary material for: Genetic diversity of a recovering European roller (Coracias garrulus) population from Serbia
Source: PLoS One. 2024 Aug 8;19(8):e0308066. doi: 10.1371/journal.pone.0308066 (PMC11309509; doi:10.1371/journal.pone.0308066)
Supplement: S4 Table — (PDF) [file pone.0308066.s012.pdf]

Table S4 Genetic variability for detected genetic clusters A, B and C in European roller (*Coracias garrulus*) from Serbia.

Pop	Locus	N	Na	Ho	He	F_{IS}
**A**	HvoB1	4	1.170	0.115	0.145	0.211
	TG04-012	6	1.330	0.126	0.248	0.495
	TG04-061	10	5.476	0.519	0.817	0.369
	TG03-098	7	5.376	0.550	0.814	0.329
	TG02-078	5	3.108	0.640	0.678	0.061
	TG03-002	8	1.454	0.214	0.312	0.320
	SAP47	5	4.163	0.670	0.760	0.123
	TG08-24	7	4.439	0.392	0.775	0.497
	TG01-040	8	4.278	0.327	0.766	0.576
	**Mean**	**6.667**	**3.422**	**0.395**	**0.591**	**0.336**
**Pop**	**Locus**	**N**	**Na**	**Ho**	**He**	F_{IS}
**B**	HvoB1	2	1.022	0.021	0.021	-0.000
	TG04-012	5	1.388	0.149	0.279	0.475
	TG04-061	8	5.461	0.660	0.817	0.203
	TG03-098	7	4.599	0.636	0.783	0.197
	TG02-078	5	3.105	0.523	0.678	0.239
	TG03-002	3	1.217	0.152	0.178	0.157
	SAP47	5	3.978	0.667	0.749	0.120
	TG08-24	5	2.803	0.213	0.643	0.675
	TG01-040	5	3.053	0.277	0.672	0.595
	**Mean**	**5**	**2.958**	**0.366**	**0.536**	**0.326**
**Pop**	**Locus**	**N**	**Na**	**Ho**	**He**	F_{IS}
**C**	HvoB1	4	1.200	0.137	0.167	0.185
	TG04-012	7	3.864	0.431	0.741	0.425
	TG04-061	9	4.596	0.548	0.782	0.306
	TG03-098	7	3.866	0.412	0.741	0.450
	TG02-078	5	2.865	0.580	0.651	0.116
	TG03-002	6	2.011	0.110	0.503	0.784
	SAP47	5	3.072	0.653	0.674	0.039
	TG08-24	8	3.903	0.370	0.744	0.507
	TG01-040	7	3.798	0.397	0.737	0.466
	**Mean**	**6.037**	**3.207**	**0.404**	**0.637**	**0.373**

N – number of alleles; Na – effective number of alleles; He – expected heterozygosity; Ho – observed heterozygosity; F_{IS} – coefficient of inbreeding.
