# Supplementary material for: Genetic diversity of a recovering European roller (Coracias garrulus) population from Serbia
Source: PLoS One. 2024 Aug 8;19(8):e0308066. doi: 10.1371/journal.pone.0308066 (PMC11309509; doi:10.1371/journal.pone.0308066)
Supplement: S5 Table — (PDF) [file pone.0308066.s013.pdf]

Table S5 Effective population size (Ne) based on linkage disequilibrium approach for European roller (*Coracias garrulus*) population from Serbia and for each of genetic cluster detected.

**Population**	**n**	**0.05**	**0.02**	**0.01**
Whole dataset	224	323.1 (206.9-581.6)	410.9 (274.6-701.9)	203.7 (119.9-394.4)
Cluster A	104	670.4 (237.1 - ∞)	183.4 (98.8-497.5)	189.6 (105.0-482.6)
Cluster B	47	98.4 (47.5-413.8)	115.3(53.8-676.1)	100.0 (14.1-481.9)
Cluster C	73	148.7 (81.2-406.1)	149.6 (93.5-293.1)	158.6 (96.6-331.5)

N_e values are given for three different frequency thresholds (0.05, 0.20, 0.01), while n is the number of individuals in each dataset. The values in brackets represent the 95% confidence interval.
