# Supplementary material for: Genetic diversity of a recovering European roller (Coracias garrulus) population from Serbia
Source: PLoS One. 2024 Aug 8;19(8):e0308066. doi: 10.1371/journal.pone.0308066 (PMC11309509; doi:10.1371/journal.pone.0308066)
Supplement: S6 Table — (PDF) [file pone.0308066.s014.pdf]

S6 Table. List of mtDNA control region haplotypes detected in 329 sequences of Coracias garrulus, with frequency and sampling locality for each haplotype. Haplotypes detected in the sample in this study are divided from those retrieved from GenBank (Nebel et al. 2019). Haplotype names correspond to those used in Figure 2 and follows those adopted from Nebel et al. (2019). Samples from this study sharing the haplotypes with those identified in Nebel et al. (2019) are shown in italics.

Accession number	Haplotype	Sampling locality	Frequency	Subspecies
		**Nebel et al. 2019**		
MK165346	Cogar1	Mosul, Iraq (1)	1	*semenowi*
MK165347	Cogar2	Lenkoran, Azerbaijan (1)	1	*semenowi*
MK165348	Cogar3	Lenkoran, Azerbaijan (1)	1	*semenowi*
MK165349	Cogar4	Lenkoran, Azerbaijan (1)	1	*semenowi*
MK165350	Cogar5	Kerman, Iran (1)	1	*semenowi*
MK165351	Cogar6	Teheran, Iran (1)	1	*semenowi*
MK165352	Cogar7	Mosul, Iraq (1)	1	*semenowi*
MK165353	Cogar8	Buxoro, Uzbekistan (1) Teheran, Iran (1), *Rtkovo, Serbia (4)*	6	*garrulus, semenowi*
MK165354	Cogar9	Nógrádszakál, Hungary (1)	1	*garrulus*
MK165355	Cogar10	Prater, Vienna, Austria (1) Südoststeiermark Styria Austria (46)	47	*garrulus*
MK165356	Cogar11	Reinprechtspölla, Lower Austria, Austria (1)	1	*garrulus*
MK165357	Cogar12	Srebarna, Silistra, Bulgaria (1)	1	*garrulus*
MK165358	Cogar13	Mannswörth, Lower Austria, Austria (1*), Subotica, Vršac, Serbia (3)*	4	*garrulus*
MK165359	Cogar14	Tabernas, Almería, Spain (2)	2	*garrulus*
MK165360	Cogar15	Tresnjevac, Vojvodina, Serbia (1) Hódmezővásárhely, Hungary (1), Sombor, Serbia (3)	5	*garrulus*
MK165361	Cogar16	Südoststeiermark, Styria, Austria (1)	1	*garrulus*
MK165362	Cogar17	Tabernas, Almería, Spain (1)	1	*garrulus*
MK165363	Cogar18	Szeged, Tolpel, Hungary (1), Subotica, Serbia (1)	2	*garrulus*
MK165364	Cogar19	Fülöpháza, Hungary (1)	1	*garrulus*
MK165365	Cogar20	Südoststeiermark, Styria, Austria (6) Widin, Bulgaria (1), *Sombor, Novo Miloševo, Novi Bečej, Subotica, Melenci, Serbia (13)*	20	*garrulus*
MK165366	Cogar21	Bükki National Park, Hungary (1)	1	*garrulus*
MK165367	Cogar22	Milas, Mugla, Turkey (1)	1	*garrulus*
MK165368	Cogar23	Szabadszállás, Hungary (1), *Bašaid, Serbia (1)*	2	*garrulus*
MK165369	Cogar24	Tabernas, Almería, Spain (1)	1	*garrulus*
MK165370	Cogar25	Tabernas, Almería, Spain (1)	1	*garrulus*

MK165371	Cogar26	Gárdony, Hungary (1), *Mokrin, Jazovo, Serbia (3)*	4	*garrulus*
MK165372	Cogar27	Adony, Hungary (1), *Rusanda, Serbia (2)*	3	*garrulus*
MK165373	Cogar28	Zdanice, Czech Republic (1), *Mokrin, Rusanda, Serbia (3)*	4	*garrulus*
MK165374	Cogar29	Tabernas, Almería, Spain (1)	1	*garrulus*
MK165375	Cogar30	Tabernas, Almería, Spain (1)	1	*garrulus*
MK165376	Cogar31	Tabernas, Almería, Spain (1)	1	*garrulus*
MK165377	Cogar32	Obersiebenbrunn, Lower Austria, Austria (1), *Stanišić, Serbia (4)*	5	*garrulus*
MK165378	Cogar33	Südoststeiermark, Styria, Austria (5), *Šaponje, Crna Bara, Mokrin, Serbia (9)*	14	*garrulus*
MK165379	Cogar34	Male Pijace, Vojvodina, Serbia (1)	1	*garrulus*
MK165380	Cogar35	Csanádi-Puszták, Hungary (1), *Ridjica, Sombor, Bašaid, Arača, Serbia (9)*	10	*garrulus*
MK165381	Cogar36	Horgos, Vojvodina, Serbia (1)	1	*garrulus*
MK165382	Cogar37	Ócsa, Hungary (1), *Ljubičevac, Vincaid, Serbia (6)*	7	*garrulus*
MK165383	Cogar38	Göttlesbrunn, Austria (1)	1	*garrulus*
MK165384	Cogar39	Bucak, Burdur, Turkey (1)	1	*garrulus*
MK165385	Cogar40	Hitzendorf, Styria, Austria (1), Hevesi-sík, Hungary (1), Dobrudscha, Constanta, Romania (1), Zimonjic, Vojvodina, Serbia (1), Kanjiza, Vojvodina, Serbia (1),Inece, Kirklareli, Turkey (1), *Novi Bečej, Rančevo, Stanišić, Hajdukovo, Subotica, Melenci, Araca, Serbia(14)*	20	*garrulus*
MK165386	Cogar41	Austria	1	*garrulus*
		**This study**		
PP783663	Cogar42	Novo Miloševo, Serbia (3)	3	*garrulus*
PP783664	Cogar43	Novo Miloševo, Serbia (1)	1	*garrulus*
PP783665	Cogar44	Novo Miloševo, Mokrin, Serbia (8)	8	*garrulus*
PP783666	Cogar45	Kumane, Novi Bečej, Siget, Novi Kneževac, Serbia(8)	8	*garrulus*
PP783667	Cogar46	Novi Bečej, Serbia (1)	1	*garrulus*
PP783668	Cogar47	Taraš, Serbia (2)	2	*garrulus*
PP783669	Cogar48	Novi Bečej, Serbia (3)	3	*garrulus*
PP783670	Cogar49	Rtkovo, Serbia (3)	3	*garrulus*
PP783671	Cogar50	Rtkovo, Stanišić, Serbia (8)	8	*garrulus*
PP783672	Cogar51	Srbovo, Serbia (5)	5	*garrulus*
PP783673	Cogar52	Srbovo, Serbia (7)	7	*garrulus*

PP783674	Cogar53	Ljubičevac, Velesnica, Serbia (7)	7	*garrulus*
PP783675	Cogar54	Velesnica, Stanišić, Serbia (5)	5	*garrulus*
PP783676	Cogar55	Velesnica, Rtkovo, Serbia (3)	3	*garrulus*
PP783677	Cogar56	Rtkovo, Serbia (1)	1	*garrulus*
PP783678	Cogar57	Rtkovo, Serbia (3)	3	*garrulus*
PP783679	Cogar58	Korbovo, Serbia (2)	2	*garrulus*
PP783680	Cogar59	Velesnica, Ljubičevac, Serbia (4)	4	*garrulus*
PP783681	Cogar60	Velesnica, Serbia (1)	1	*garrulus*
PP783682	Cogar61	Ljubičevac, Serbia (2)	2	*garrulus*
PP783683	Cogar62	Srbovo, Serbia (4)	4	*garrulus*
PP783684	Cogar63	Kruševlje, Sombor, Serbia (5)	5	*garrulus*
PP783685	Cogar64	Ridjica, Arača, Serbia (3)	3	*garrulus*
PP783686	Cogar65	Sombor, Stanišić, Ridjica, Serbia (3)	3	*garrulus*
PP783687	Cogar66	Sombor, Stanišic, Serbia (4)	4	*garrulus*
PP783688	Cogar67	Idjoš, Mokrin, Rusanda, Serbia (7)	7	*garrulus*
PP783689	Cogar68	Idjoš, Aradac, Serbia (6)	6	*garrulus*
PP783690	Cogar69	Crna Bara, Serbia (1)	1	*garrulus*
PP783691	Cogar70	Mokrin, Novi Kneževac, Crna Bara, Serbia (3)	3	*garrulus*
PP783692	Cogar71	Mokrin, Serbia (2)	2	*garrulus*
PP783693	Cogar72	Jazovo, Serbia (1)	1	*garrulus*
PP783694	Cogar73	Mokrin, Serbia (1)	1	*garrulus*
PP783695	Cogar74	Subotica, Serbia (1)	1	*garrulus*
PP783696	Cogar75	Subotica, Serbia (1)	1	*garrulus*
PP783697	Cogar76	Subotica, Serbia (1)	1	*garrulus*
PP783698	Cogar77	Beljina, Serbia (4)	4	*garrulus*
PP783699	Cogar78	Boždarevac, Serbia (3)	3	*garrulus*
PP783700	Cogar79	Aradac, Serbia (3)	3	*garrulus*
PP783701	Cogar80	Aradac, Serbia (3)	3	*garrulus*
PP783702	Cogar81	Aradac, Serbia (1)	1	*garrulus*
PP783703	Cogar82	Vršac, Serbia (3)	3	*garrulus*
PP783704	Cogar83	Arača, Serbia (1)	1	*garrulus*
PP783705	Cogar84	Melenci, Serbia (2)	2	*garrulus*
PP783706	Cogar85	Arača, Slano Kopovo, Serbia (5)	5	*garrulus*
PP783707	Cogar86	Arača, Serbia (1)	1	*garrulus*

PP783708	Cogar87	Rusanda, Serbia (1)	1	*garrulus*
PP783709	Cogar88	Vincaid, Serbia (2)	2	*garrulus*
